# Ovarian Cancer Previvors: How to manage these patients?

**DOI:** 10.6061/clinics/2019/e1343

**Published:** 2019-07-17

**Authors:** Jesus Paula Carvalho, Edmund Chada Baracat, Filomena Marino Carvalho

**Affiliations:** I Disciplina de Ginecologia, Instituto do Cancer do Estado de Sao Paulo (ICESP), Hospital das Clinicas HCFMUSP, Faculdade de Medicina, Universidade de Sao Paulo, Sao Paulo, SP, BR; II Departamento de Patologia, Faculdade de Medicina FMUSP, Universidade de Sao Paulo, Sao Paulo, SP, BR

Previvors are individuals who have a much greater predisposition to cancer than individuals in the general population but who have not yet developed the disease. This group comprises individuals with deleterious mutations, family histories of cancer, and other high-risk factors for cancer 1. Preventive strategies targeting previvors correspond to the earliest measure of cancer prevention 2,3. Interventions addressed to previvors are more efficient in the sense that a greater benefit can be observed per individual receiving the intervention 2. Ovarian cancer is the most lethal of all gynecological malignancies, and screening programs do not significantly decrease mortality from this disease, although previvors can currently be well identified 4-7. Women with germline pathogenic mutations of the *BRCA1* and/or *BRCA2* genes have a lifetime risk of ovarian cancer development that ranges from 20% to 65% 8-10. For these ovarian cancer previvors, the concept of taking action to avoid cancer is incipient, and well-structured strategies and programs are lacking 11,12.

## The origin of precursor lesions and ovarian cancer

High-grade serous ovarian carcinoma (HGSC), the most frequent and aggressive histological type of ovarian cancer, originates in fimbrial cells that are secondarily implanted in the ovary 13. This type of carcinoma can start in the fallopian tubes and implant and grow in the ovary at an early stage, or it can originate in normal tubal cells implanted in the ovulatory wound during ovulation 13. The origin of cancer depends on local factors and/or host fragility 14,15. Women with mutations in the *BRCA* genes carry conditions that favor the development of HGSC in the tubal epithelium 15. It is therefore reasonable to consider that efforts to prevent ovarian cancer should focus on intervention in the fimbriae to prevent cell implantation in the ovaries, and thus, cancer development.

Factors that prevent fimbrial cell implantation in the ovary, such as the prolonged use of anovulatory contraceptives and salpingectomy for any reason, have been shown to significantly reduce the incidence of ovarian cancer 16,17. The implantation of fimbrial cells in the ovary begins with the onset of ovulation. The amount of ovulation occurring over a woman's lifetime is closely related to the incidence rate of ovarian cancer 18,19. It is reasonable to suppose that earlier interruptions of fimbrial cell implantation will have a greater benefit in the prevention of ovarian cancer.

**Figure 1:**
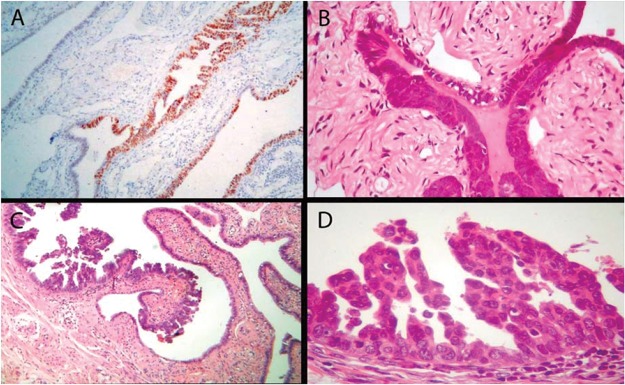
Spectrum of tubal fimbrial lesions. (A) Epithelial cells expressing p53 (p53 signature), (B) hyperplastic tubal epithelium with atypia, (C) and (D) STIC.

Precursor lesions and early serous carcinoma were first identified in specimens from prophylactic salpingo-oophorectomies performed in high-risk patients 20,21. Examples of these precursors, which precede the onset of invasive ovarian cancer by several years, are the p53 protein signature, atypia in hyperplasic epithelium, and serous tubal intraepithelial carcinoma (STIC) 21 (Figure 1). Precursor cancer is a definable pathological state that progresses to cancer and can be targeted to prevent cancer progression 2. The HGSC precursor, termed the p53 signature, precedes invasive ovarian carcinoma by decades, and STIC precedes carcinoma by at least 6 years 21-23. Therefore, acting on these precursors seems to be a good strategy to prevent HGSC, but this strategy should be implemented before the age at which prophylactic surgery has to been performed.

## Ovarian cancer in previvors in the genetic testing era

Many factors have contributed to the increased identification of ovarian cancer previvors, including a) the advent and popularization of genetic testing, b) increased interest in the identification of ovarian carcinoma survivors with pathogenic mutations who are candidates for targeted therapy with poly(ADP-ribose) polymerase (PARP) inhibitors, and c) the identification of the relatives of these patients who also carry pathogenic mutations 24-26. The major question is, what measures should we take with these ovarian cancer previvors beyond the recommended prophylactic salpingo-oophorectomy, which has undesirable consequences?

Prophylactic salpingo-oophorectomy is currently recommended for women with deleterious *BRCA1* mutations at 35-40 years of age (or at any time after childbearing is completed). For those with deleterious *BRCA2* mutations, this surgery is recommended at 40-45 years of age 27,28. However, the performance of prophylactic surgery at the recommended age confers protection in approximately 80-90% of cases 29-31. In addition, a considerable number of women already present with occult lesions at the time of surgery, and the disease may develop in the peritoneum. Hidden serous carcinoma or STIC has been detected in 1-17% of surgical specimens obtained during such surgeries 32,33.

In a prospective study that followed 5,783 women with a *BRCA1* or *BRCA2* mutation for 5.6 years, 186 ovarian, fallopian, and peritoneal cancers were observed in addition to the 46 occult cancers observed at the time of salpingo-oophorectomy and the 32 peritoneal cancers observed after oophorectomy 34. In this study, the specimens from the risk reduction surgeries performed in the patients with deleterious *BRCA1* mutations, the detection frequency of occult carcinoma varied with age (1.5% at <40 years old, 3.8% at 40-49 years old, and over 7% at >50 years old, reaching 12% at 60-64 years old) 34. The occurrence rate of peritoneal carcinoma after salpingo-oophorectomy in patients with deleterious *BRCA1/BRCA2* mutations ranged from 0.8% to 1.8% 30,35-37. Moreover, most women do not undergo prophylactic salpingo-oophorectomy at the recommended age; approximately only 17% undergo surgery before the age of 40 years old 38.

Prophylactic salpingo-oophorectomy has a great impact on women's quality of life, as it results in premature menopause, vasomotor symptoms, sexual dysfunction, cardiovascular disease, osteoporosis, cognitive deficits, and an increased risk of premature death 39-46. These undesired effects are the main barriers to patients' adherence to these prophylactic procedures, even in the face of the great risk of ovarian cancer 47,48.

## Prophylactic salpingectomy with delayed oophorectomy

An alternative to prophylactic salpingo-oophorectomy, which does not result in early menopause, is prophylactic salpingectomy with delayed oophorectomy. The theoretical benefit of this procedure is based on the expectation that early removal of the fallopian tubes prevents the implantation of fimbrial cells in the ovulatory wound. Thus, early salpingectomy provides greater protection than late salpingectomy.

Bilateral salpingectomy with delayed oophorectomy is less damaging to women than salpingo-oophorectomy; consequently, this procedure has a greater the chance to be accepted by women 9,49-53. A prospective, nonrandomized, pilot study with 43 premenopausal patients with a *BRCA* mutation was conducted, and 19 (44%) patients chose salpingectomy with delayed oophorectomy, 12 (28%) patients chose salpingo-oophorectomy, and 12 (28%) patients chose to be screened only 52. The patients who underwent salpingectomy were satisfied with their choice and had decreased worry and anxiety about cancer after the surgery. In a qualitative study performed with 39 *BRCA1/2* mutation carriers and 23 health professionals using explorative interviews, the maintenance of ovarian function with the delay of the negative effects of early menopause and infertility was considered a facilitator influencing the choice to undergo salpingectomy with delayed oophorectomy instead of salpingo-oophorectomy by both patients and treating professionals 50. On the other hand, the seriousness of ovarian cancer and the lack of strong evidence for the new strategy worries professionals and patients 50,51. However, although there are no large prospective randomized studies indicating increased safety and a reduced risk of ovarian cancer with salpingectomy compared to the safety and risk with oophorectomy, we have some promising evidence supporting this concept. A simulation model was developed to estimate the costs and benefits of the following three risk-reducing strategies in *BRCA* mutation carriers: bilateral salpingo-oophorectomy at 40 years old, bilateral salpingectomy at 40 years old, and bilateral salpingectomy at 40 years old followed by bilateral oophorectomy at 50 years old 54. Although bilateral salpingo-oophorectomy was associated with the greatest risk reduction for ovarian cancer, when quality of life was included in the model, bilateral salpingectomy with delayed oophorectomy proved to be an acceptable alternative for those unwilling to undergo the first procedure 54.

The proportion of patients who choose delayed oophorectomy suggests that patient accrual for a clinical trial of prophylactic salpingectomy with delayed oophorectomy is possible.

## Preimplantation genetic diagnosis (PGD)

Women with pathogenic mutations related to ovarian cancer have a high risk of developing ovarian cancer and may also transmit these mutations to their offspring. Current genetic testing enables the identification of pathogenic germline mutations in women of any age and in embryos.

Preimplantation genetic diagnosis (PGD) is considered to be an acceptable intervention to prevent the transmission of deleterious genetic conditions to the next generation, although much ethical controversy surrounds the consideration of which deleterious conditions should be deemed serious enough to justify such an intervention 55-57. PGD decisions are complex, as reflected in a study conducted in Israel with 70 women with deleterious *BRCA1/2* mutations, for whom the possibility of preventing the transmission of these mutations through PGD and IVF was offered at no cost. Only 25.7% of patients accepted the proposal, and acceptance had no relationship to age or religious beliefs 58.

The combined performance of early prophylactic salpingectomy, oocyte uptake, IVF, and, when acceptable, PGD, would have many advantages over oophorectomy, at least from a theoretical point of view. First, it would not only preserve ovarian function but also enable pregnancy with oocyte uptake and IVF. The need for IVF, in turn, presents a great opportunity for PGD. Considering that precursors (the p53 signature and STIC) predate ovarian cancer by decades or years, this strategy could provide an opportunity for early intervention. Moreover, salpingectomy could even be performed in young women with pathogenic mutations before they first ovulate. This strategy, combined with oocyte uptake, IVF, and PGD, would allow the implantation of healthy embryos in mothers at a reduced risk of ovarian cancer while preventing infertility due to premature ovarian failure and/or early menopause 59,60. Women adhering to this strategy would have more time for oocyte capture and the growth of healthy embryos for future implantation than would those expecting through natural pregnancies.

## Consequences of the proposal and discussion

When considered alone, many concepts, such as prophylactic salpingo-oophorectomy, prophylactic salpingectomy with delayed oophorectomy, oocyte uptake, IVF, PGD, ovarian failure, and premature menopause, may be difficult for laypeople to understand. A strategy combining all of these concepts, named “the maximal effort to prevent ovarian cancer while preserving the ovaries,” should be proposed to high-risk patients and might be better accepted by a large number of women.

This strategy could be tested in a randomized, global study that addresses all aspects of this complex issue from the perspectives of oncology, human reproduction, genetics, legality, medical ethics, and relevant educational, cultural, psychosocial, and religious aspects.
